# Chronic granulomatous disease and McLeod syndrome: Stem cell transplant and transfusion support in a 2-year-old patient—a case report

**DOI:** 10.3389/fimmu.2022.994321

**Published:** 2022-08-23

**Authors:** Louise Helander, Chris McKinney, Kathleen Kelly, Samantha Mack, Mary Sanders, Janice Gurley, Larry J. Dumont, Kyle Annen

**Affiliations:** ^1^ ClinImmune Cell and Gene Therapy, Department of Medicine, University of Colorado Anschutz School of Medicine, Denver, CO, United States; ^2^ Transfusion Medicine and Apheresis, Department of Pathology, Children’s Hospital Colorado, Denver, CO, United States; ^3^ Blood and Marrow Transplant Therapy Program, Children’s Hospital Colorado, Denver, CO, United States; ^4^ Vitalant Research Institute, Vitalant, Denver, CO, United States; ^5^ Department of Pathology, University of Colorado Anschutz School of Medicine, Denver, CO, United States

**Keywords:** case report, McLeod, chronic granulomatous disease, pediatrics, allogeneic stem cell transplant, transfusion

## Abstract

Chronic granulomatous disease (CGD) with McLeod neuroacanthocytosis syndrome (MLS) is a contiguous gene deletion disorder characterized by defective phagocytic function and decreased Kell antigen expression. CGD cure is achieved through hematopoietic stem cell transplant (HSCT) usually in the peri-pubescent years. The presence of MLS makes peri-transfusion support complex, however. Herein, we present the youngest known case of HSCT for CGD in the setting of MLS. A 2-year-old male patient was diagnosed with CGD plus MLS. Due to the severity of the child’s systemic fungal infection at diagnosis, HSCT was deemed the best treatment option despite his small size and age. A related, matched donor was available, and a unique red blood cell support plan had been implemented. Reduced-intensity conditioning was used to reduce the transplant-related mortality risk associated with myeloablative protocols. The transplant course was uneventful; autologous red blood cell (RBC) transfusion support was successful and allowed for the avoidance of possible antibody formation if allogeneic units had been used. The patient achieved 1-year relapse-free survival. The developed protocols provide a viable path to transplant in the very young, and early transplant to cure could reduce disease-related morbidity.

## Introduction

The most common form of chronic granulomatous disease (CGD) is the result of pathologic variation in the *CYBB* gene located on the short arm of the X-chromosome (1). Adjacent to the *CYBB* gene is the *XK* gene, whose concurrent deletion can result in the contiguous gene deletion disorder, McLeod neuroacanthocytosis syndrome (MLS) (1, 2). Together, these syndromes occur almost exclusively in male individuals and are characterized by recurrent bacterial and fungal infections due to defective phagocyte function, decreased expression of the Kell antigen system, which is covalently linked to the *XK* gene product (Kx) on red blood cells (RBC), and late-onset neurodegenerative sequelae (1, 3, 4).

Currently, the only cure for CGD is allogeneic hematopoietic stem cell transplant (HSCT) (5). Timing to transplant remains center dependent and is based on factors including patient size, donor graft source and availability, and the health of the patient (6). Improvement in antibiotic and antimycotic prophylactic regimens has improved patient survival, allowing HSCT to be delayed until patients develop infections that are chronic or refractory to medical management (5, 7). MLS adds a layer of complexity when considering HSCT. Transfusion support is required peri-transplant, which can result in the development of clinically significant alloantibodies (anti-Kx and anti-Km) (8) due to the patient’s lack of the Kx and reduced Kell antigen expression, antigens almost universally found on donor blood products (9).

We report on the youngest known case of related allogeneic HSCT to cure CGD in a male patient with CGD and MLS and the unique strategies used to achieve successful transplant with alloimmunization avoidance.

## Case report

Following a history of multiple infections starting 2 months after birth, including ruptured appendicitis at 22 months, a 2-year-old male patient presented with new-onset Nocardia lymphadenitis and multiple cavitary lung nodules. CGD was diagnosed following an abnormal oxidative burst assay using dihydrorhodamine. Subsequent chromosomal microarray studies confirmed the loss of a 1.1-Mb region in Xp21.1-p11.4. The deleted region included *XK* and the *CYBB* genes, confirming the diagnosis of X-linked CGD with MLS. Following maternal testing, this was determined to be a *de novo* mutation. Due to the severity of the patient’s immunodeficiency and disseminated Nocardia infection, allogeneic HSCT was deemed the best option for long-term survival (5).

An unaffected sibling was identified as a 10/10-matched, ABO-matched donor. The patient had remained transfusion naïve throughout his life with negative antibody screens. The hospital transfusion service was consulted early in treatment planning to determine the best transfusion strategy peri-transplant. The decision was made to contact the rare blood donor program in an attempt to find CGD with MLS-compatible RBC units to avoid possible sensitization risk to the Kell blood group system. The transfusion service was notified that compatible RBC units were not currently available and a suitable donor could not be identified. The risk of alloimmunization from non-MLS compatible units could complicate the post-transplant course (9–11), so an autologous whole blood donation program was developed by the transfusion service in conjunction with a local blood supplier to collect and freeze autologous units for a child of this size (~11 kg).

Four autologous 110-ml (≤10.5 ml/kg) whole blood donations were performed over 15 weeks at a pediatric in-hospital blood donation center. Pre-donation hemoglobin and hematocrit were checked either through the core lab or using the HemoCue (Brea, CA, USA). Samples from each donation were cultured: aerobic (0.5 ml), anaerobic (3 ml), and fungal (1.5 ml). Fungal testing was completed out of an abundance of caution due to the patient’s systemic nocardiosis, and the samples were held for 21 days to ensure adequate time for growth. The remaining approximately 100 ml of blood was labeled following standard International Society of Blood Transfusion (ISBT) guidelines and sent to an outside facility for processing, freezing, and storage using the Haemonetics ACP-215 (small-volume autologous red blood cell collection procedure previously documented (12)).

**Figure 1:**
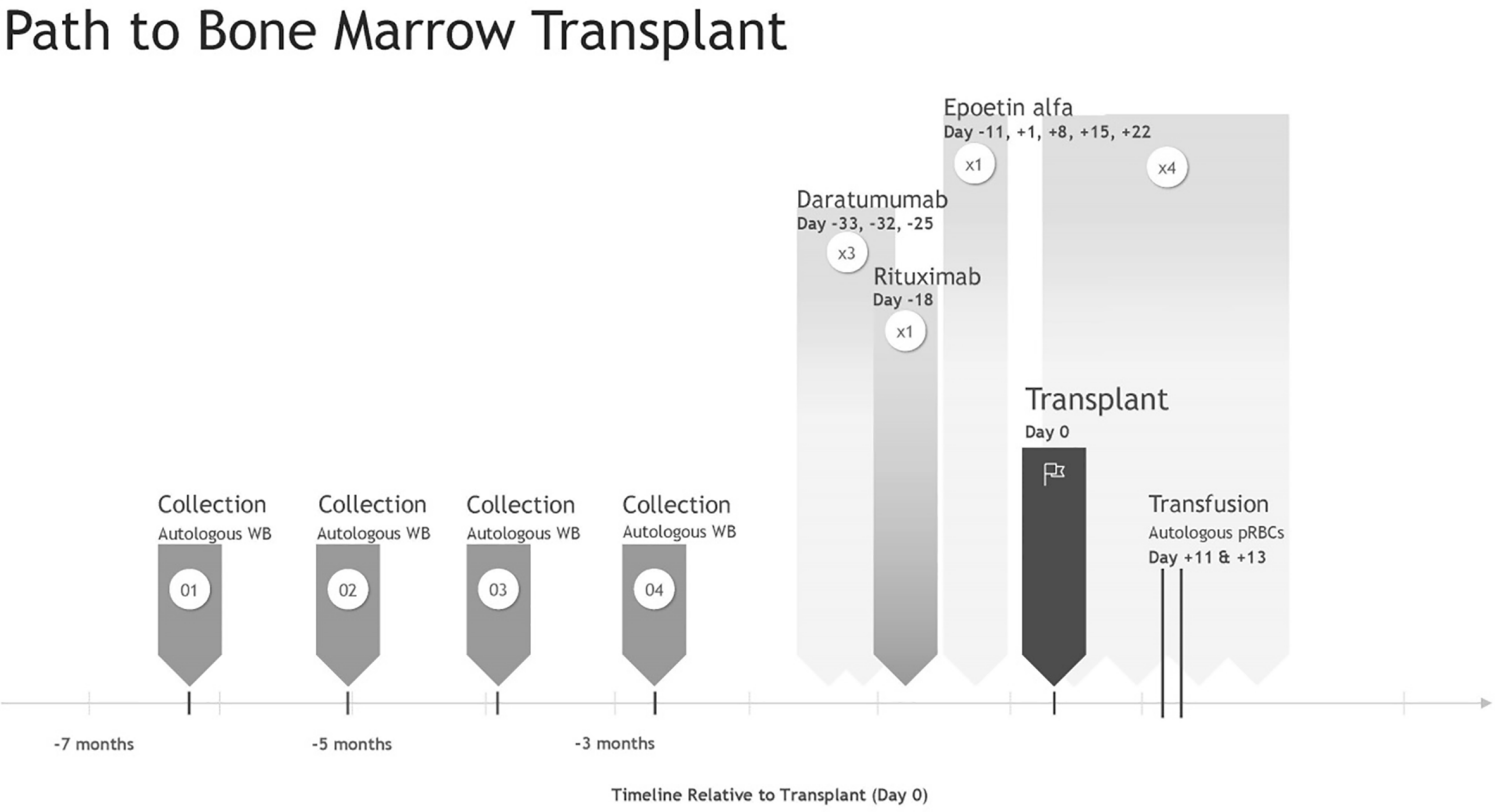
Patient pre and post transplant timeline relative to Day 0.

The preparatory transplant regime included daratumumab (×3 doses) and rituximab (×1 dose) to reduce the risk of RBC alloimmunization if autologous transfusion failed and during stem cell transplant. This was followed by reduced-intensity non-myeloablative chemotherapy modeled after recommendations from the 2014 reduced-intensity conditioning (RIC) study for human leukocyte antigen (HLA)-matched HSCT in patients with CGD, including high-dose fludarabine (30 mg/m^2^/dose; one dose per day on day −7 to −2), intravenous busulfan (area under the curve: 63.2 mgL × h; days −5 to −3), and rabbit-ATG (2.5 mg/kg one dose per day on days −4 to −2) (13). The bone marrow harvested graft was processed through the Optia Apheresis System (Terumo BCT, Lakewood, CO, USA) to reduce the allogeneic RBC burden. Erythropoietin (100 U/kg/dl) and iron supplementation were administered 7 days prior to HSCT to increase autologous RBC volume. On day +1 post-transplant, weekly recombinant human erythropoietin (rEPO) (600 U/kg/dl) was initiated × 4 weeks to accelerate RBC engraftment (**Figure 1**). The patient’s post-transplant RBC transfusion threshold was reduced to ≤6.0 g/dl while asymptomatic in an attempt to reduce transfusion frequency. Phlebotomy for laboratory testing was reduced to every other day to moderate iatrogenically induced anemia.

Two 5-ml/kg autologous RBC transfusions were required on day +11 and day +13 post-transplant (Hb < 6.0 g/dl). Autologous RBC units were thawed using a Haemonetics protocol modified for small volumes before transport to the pediatric hospital blood bank. As the Haemonetics ACP-215 is a closed system, units were labeled with a 2-week expiration date. Transfusion was completed following standard hospital blood infusion protocols. Both were tolerated without reaction, and on day +15, hemoglobin values began to increase. Platelet support was not necessary peri-transplant.

**Figure 2:**
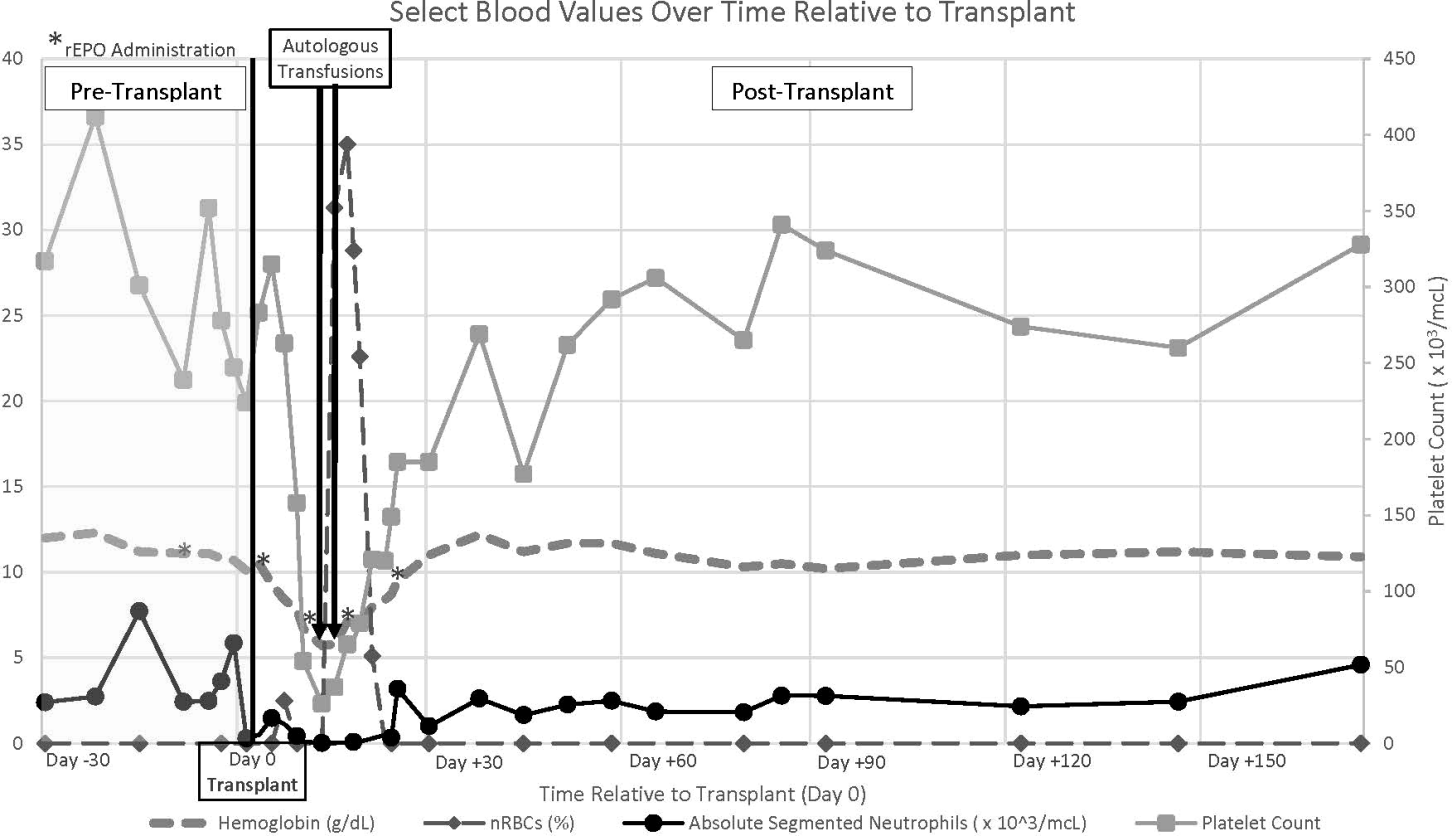
Select blood count values pre and post-transplant relative to Day 0.

Neutrophil engraftment occurred by 23 day post-transplant (**Figure 2**), and normal neutrophil oxidative burst was demonstrated by dihydrorhodamine assay. A PCR-based short tandem repeat sorted chimerism assay on day +64 indicated complete donor cell engraftment of the myeloid cell lineage with incomplete engraftment of the lymphoid lineage (21%). The patient remains free from relapse over 1-year post-transplant with continued mixed chimerism of the lymphoid cell line (65%) and no evidence of graft-versus-host disease (GVHD). The patient has discontinued anti-microbial prophylaxis and begun vaccine re-immunization.

## Discussion

In the past, most successful HSCT in patients with CGD has been achieved using HLA-matched related donors in conjunction with myeloablative conditioning regimes (5, 7). RIC has been previously explored with mixed results (14, 15). Due to the patient’s ongoing Nocardia infection, the increased requirement for blood transfusions, and the transplant-related mortality (TRM) risk associated with myeloablative regimes (5), a RIC program was chosen.

A RIC regime was not without risk: recipient plasma cells are known to survive RIC protocols (16). Post-transplant, these recipient plasma cells may continue to produce antibodies if previously sensitized resulting in delayed or even failed engraftment or can become sensitized post-transplant due to the survival and priming of recipient T cells either during transfusion or in response to the graft (10). Kordes et al. previously reported on a case with this suspected mechanism (10). Late post-transplant hemolysis, suspected from the formation of anti-Kx and anti-Kell antibodies by non-ablated recipient B-cells, led to chronic, life-threatening hemolysis requiring years-long transfusion support. Allogenic transfusion of our patient peri-transplant would have exposed him to Kx and Kell antigens, putting him at risk for similar antibody formation. The antigens found on the matched bone marrow transplant (BMT) also present an alloimmunization risk from the Kx and Kell antigens.

A pre-conditioning regime using rituximab to deplete B cells and daratumumab to inhibit plasma cells was used to try to blunt the immune antibody response and reduce alloimmunization risk (11). rEPO was used early to increase hematocrit during conditioning as well as enhance repopulation of the erythroid cell line post-allogeneic transplant (17). Four autologous RBC components were collected over 15 weeks to ensure an adequate number of available units. The strategy to freeze autologous RBC units for peri-transplant pediatric RBC support has been used previously (15), but never on a child of this size (~11 kg). Transfusion in this case was uneventful with no documented hemolysis and no known antibody formation to date.

The transplant course was uneventful. Hematologic reconstitution occurred promptly, and alloimmunization was not observed. While it is unclear which modifications to the standard transplant course contributed most to this success and this has only been completed on a single patient, this nonetheless demonstrates that CGD with McLeod patients can be safely and successfully transplanted much earlier than currently practiced.

The development of an autologous red cell unit collection procedure for the very small could serve a wider population. Small patients with rare blood types, including Bombay or k-antigen negative phenotypes, could benefit from being able to self-donate and cryopreserve units for future procedures, including transplants, to avoid future alloimmunization. This could be an important alternative RBC source for these patients, as the rare blood registry does not always have compatible units available, as in our case, and may not provide them to an unsensitized individual in an effort to protect the resource. Autodonation could also prove beneficial for patient groups with partial Rh blood group system antigens whose only antigen matches may be family members. Once rare blood group antibodies have formed, autocollection may be the only option for compatible units, but this option has traditionally been relegated to individuals over 110 pounds. This protocol would provide alternatives to avoid further alloimmunization and possibly life-threatening hemolytic events.

## Data availability statement

The original contributions presented in the study are included in the article/Supplementary Material. Further inquiries can be directed to the corresponding author.

## Ethics statement

Written informed consent was obtained from the minor(s)’ legal guardian/next of kin for the publication of any potentially identifiable images or data included in this article.

## Author contributions

MS, JG, LH, and KA developed the transfusion protocol. KA oversaw the two necessary transfusions. LH wrote the paper. KK, SM, and LD developed, performed, and oversaw the operation responsible for the red blood cell freezing/thawing protocol. MS and JG performed the red blood cell collection procedures. CM developed and oversaw the transplant course. All authors contributed to the article and approved the submitted version.

## Acknowledgments

Thank you to Pepe (the vein whisperer) for his contributions to this patient’s care.

## Conflict of interest

The authors declare that the research was conducted in the absence of any commercial or financial relationships that could be construed as a potential conflict of interest.

## Publisher’s note

All claims expressed in this article are solely those of the authors and do not necessarily represent those of their affiliated organizations, or those of the publisher, the editors and the reviewers. Any product that may be evaluated in this article, or claim that may be made by its manufacturer, is not guaranteed or endorsed by the publisher.

## References

[1] Roulis E Hyland C Flower R Gassner C Jung HH Frey BM . Molecular basis and clinical overview of McLeod syndrome compared with other neuroacanthocytosis syndromes a review. JAMA Neurol (2018) 75(12):1554–62. doi: 10.1001/jamaneurol.2018.2166 30128557

[2] Watkins CE Litchfield J Song E Jaishankar GB Misra N Holla N . Chronic granulomatous disease, the McLeod phenotype and the contiguous gene deletion syndrome-a review. Clin Mol Allergy (2011) 9:13. doi: 10.1186/1476-7961-9-13 PMC3267648 22111908

[3] Denomme GA . Kell and kx blood group systems. Immunohematology (2015) 31(1):14–9. doi: 10.21307/immunohematology-2019-065 26308465

[4] Pu JJ Redman CM Visser JWM Lee S . Onset of expression of the components of the kell blood group complex. Transfusion (2005) 45:969–74. doi: 10.1111/j.1537-2995.2005.04289.x 15934996

[5] Arnold DE Heimall JR . A review of chronic granulomatous disease. Adv Ther (2017) 34(12):2543–57. doi: 10.1007/s12325-017-0636-2 PMC5709447 29168144

[6] Leiding JW Holland SM . Chronic granulomatous disease. In: Adam MP Everman DB Mirzaa GM Pagon RA Wallace SE Bean LJH , editors. GeneReviews®. Seattle (WA: University of Washington, Seattle (2012). p. 1993–2021. Available at: https://www.ncbi.nlm.nih.gov/books/. 22876374

[7] Suzuki N Hatakeyama N Yamamoto M Mizue N Kuroiwa Y Yoda M . Treatment of McLeod phenotype chronic granulomatous disease with reduced-intensity conditioning and unrelated-donor umbilical cord blood transplantation. Int J Hematol (2007) 85:70–2. doi: 10.1532/IJH9706129 17261504

[8] Storry JR . Other blood group systems and antigens. In: Fung MK Eder AF Spitalnick SL Westhoff CM , editors. Technical manual 19th ed. Bethesda: AABB (2017). p. p.319–348.

[9] Franchini M Gandini G Aprili G . Non-ABO red blood cell alloantibodies following allogeneic hematopoietic stem cell transplantation. Bone Marrow Transpl (2004) 33:1169–72. doi: 10.1038/sj.bmt.1704524. 15094753

[10] Kordes U Binder TMC Eiermann TH Hassenpflug-Diedrich B Hassan MA Beutel K . Successful donor-lymphocyte infusion for extreme immune-hemolysis following unrelated BMT in a patient with X-linked chronic granulomatous disease and McLeod phenotype. Bone Marrow Transpl (2008) 42:219–20. doi: 10.1038/bmt.2008.159. 18560413

[11] Nickel RS Flegel WA Adams SD Hendrickson JE Liang H . The impact of pre-existing HLA and red blood cell antibodies on transfusion support and engraftment in sickle cell disease after nonmyeloablative hematopoietic stem cell transplantation from HLA-matched sibling donors: A prospective, single center, observational study. E Clin Med (2020) 24. doi: 10.1016/j.eclinm.2020.100432. PMC7327930 32637902

[12] Kelly K Helander L Hazegh K Stanley C Moss R Mack S . Cryopreservation of rare pediatric red blood cells for support following bone marrow transplant. Transfus (2022) 62(5):954–60. doi: 10.1111/trf.16878. 35403731

[13] Güngör T Teira P Slatter M Stussi G Stepensky P Moshous D . Inborn errors working party of the European society for blood and marrow transplantation. reduced-intensity conditioning and HLA-matched haemopoietic stem-cell transplantation in patients with chronic granulomatous disease: a prospective multicentre study. Lancet (2014) 383(9915):436–48. doi: 10.1016/S0140-6736(13)62069-3 24161820

[14] Yu J Azar AE Chong HJ Jongco AM 3rd Prince BT . Considerations in the diagnosis of chronic granulomatous disease. J Pediatr Infect Dis Soc (2018) 7(suppl 1):S6–S11. doi: 10.1093/jpids/piy007 PMC5946934 29746674

[15] Honig M Flegel WA Schwarz K Freihorst JF Baumann U Seltsam A . Successful hematopoietic stem-cell transplantation in a patient with chronic granulomatous disease and McLeod phenotype sensitized to kx and K antigens. Bone Marrow Transpl (2010) 45:209–11. doi: 10.1038/bmt.2009.115 PMC5662187 19503108

[16] Fasano RM Mamcarz E Adams S Donohue J Sugimoto K Tian X . Persistence of recipient human leukocyte antigen (HLA) antibodies and production of donor HLA antibodies following reduced intensity allogeneic hasematopoietic stem cell transplantation. Br J Haematol (2014) 166:425–34. doi: 10.1111/bjh.12890 PMC4174569 24750103

[17] Kaesson S . Clinical use of rHuEPO in bone marrow transplantation. Med Onc (1999) 16:2–7. doi: 10.1007/BF02787351 10382935

